# A Computational Model of Tumor Growth and Anakoinosis

**DOI:** 10.3389/fphar.2019.00287

**Published:** 2019-03-26

**Authors:** Pan Pantziarka, Lina Ghibelli, Albrecht Reichle

**Affiliations:** ^1^The George Pantziarka TP53 Trust, London, United Kingdom; ^2^Anticancer Fund, Brussels, Belgium; ^3^Dipartimento di Biologia, Università di Roma Tor Vergata, Rome, Italy; ^4^Department of Hematology and Oncology, University Hospital Regensburg, Regensburg, Germany

**Keywords:** anakoinosis, computational model, cancer, drug repurposing, biomodulatory therapy

## Abstract

Anakoinosis is a new cancer treatment paradigm that posits a key role for communicative reprogramming within tumor systems. To date no mathematical or computational models of anakoinosis have been developed. Here we outline the NEATG_A system, a first computational model of communicative reprogramming. The model recapitulates key features of real tumor systems and responses to both traditional cytotoxic treatments and biomodulatory/anakoinotic treatments. Results are presented and discussed, particularly with respect to the implications for future cancer treatment protocols.

## Introduction

NEATG (Non-physiological Evolutionary Algorithm for Tumor Growth) is a computational model of tumor growth which recapitulates a number of phenomena associated with real tumor growth and response to anticancer treatments (Pantziarka, 2016). NEATG is a stochastic multi-scale agent-based system that models cell-to-cell and tissue-level interactions, population growth dynamics and cell competition. The system is structured such that different evolutionary scenarios can be developed and previous work has included the modeling of tissue homeostasis, untreated tumor growth, the impact of nutrient stress and response to cytotoxic treatments. Previous results have shown cellular growth dynamics, tissue invasion patterns and tumor regrowth following cytotoxic treatment that correspond to published data from *in vitro* growth of human cancer cell lines. Model outputs were shown to be concordant with cell growth data from a panel of established cancer cell lines. In particular the results showing accelerated tumor regrowth following aggressive cytotoxic treatment were unexpected, suggesting that cell competition and cell death were both drivers of resistance to cytotoxic cancer treatments.

Anakoinosis is a new cancer treatment paradigm predicated on a key role for communicative reprogramming of tumor systems (Hart et al., 2015). Building on a systems biology approach to cancer (Reichle and Vogt, 2008), anakoinosis utilizes a range of non-cancer and cancer drugs in combination to treat advanced disease (Walter et al., 2017). In contrast to standard therapies, anakoinosis protocols are characterized by low-toxicity and a good safety profile, with encouraging responses in a number of clinical trials to date. The use of drug repurposing, that is the use of non-cancer drugs as cancer treatments (Pantziarka et al., 2014, 2015), is especially a notable feature of this approach.

To date no mathematical or computational models of anakoinosis have been developed. NEATG_A, a new computational model, is an extension of the NEATG system to incorporate elements of cell-tissue communication such that the impact of anakoinosis can be modeled. As with the original NEATG system this is a computational model at a level of abstraction that does not include the relevant biological/molecular pathways that implement these behaviors in the patient or animal model. Neither does the model include data on the immune response, tumor neo-angiogenesis, aspects of the tumor architecture or other biologically relevant factors. Instead the focus is on abstract elements which characterize the functional behaviors of modeled cells and tissues and how these relate to cancer growth and response to treatment.

This paper outlines results which show how intrinsic communicative networks act as inherent anticancer mechanisms and that disruption of such communication promotes cancer growth and tumor invasion. Furthermore the model shows how communicative reprogramming after tumor development can impact cancer growth.

## Materials and Methods

Non-physiological Evolutionary Algorithm for Tumor Growth is a computational model coded in the Java programming language. It is an agent-based model utilizing the gridded structure and iterative processing of cellular automata and incorporating a novel genetic algorithm to generate evolutionary changes in the system. The system is non-deterministic and includes both cell-level and tissue-level behaviors.

The tissue level is represented as a rectangular grid of *m*×*n* elements, with each element hosting a number of software cells. Each grid element exists in a given state, determined by the relative population of different cell types that inhabit that cell. Grid elements can change state at each iteration (clock-ticks) of the system depending on the population of cells that it contains.

Cells are modeled as complex data structures that incorporate a number of genes, metabolic regulators, a nutrient store and an internal clock to count down its lifetime. Although the model can be used with different sized genomes, as in the previous version of NEATG the work described below uses cells with three genes as this is computationally tractable and displays sufficient levels of genetic evolution as to provide useful results. Cells exist in a given state:

$$S=\left\{{\text{HEALTHY, DIVIDING, APOPTOTIC, TO}}_{-}{\text{BE}}_{-}\text{CLEARED, NECROTIC}\right\}$$

Cells can change state in response to the availability of nutrients to meet metabolic demands and depending on genetic factors. When the number of cells in a given grid element exceeds the grid element carrying capacity (i.e., the grid element is over-crowded) cellular competition takes place and the least fit cells are removed. The system includes two classes of cells – *Normal* and *Malignant* cells. These are structurally similar and follow the same cellular life-cycle and processes, however *Malignant* cells differ in that they have the ability to both mutate and invade neighboring grid elements during cell division.

Full details of the NEATG system are described in Pantziarka (2016). As in that previous work, the experiments in this paper use a simple cell structure that consists of three genes and the same parameters as described in the Homeostasis section of the original publication. Note that the cell state *Healthy* represents the biological state of cellular *Quiescence*, it implies that the cell is in a reversible non-proliferating state (Daignan-Fornier and Sagot, 2011; Terzi et al., 2016). Where the internal model code uses the term *Healthy*, we shall use the more biologically relevant term *Quiescent* in this paper.

The principal innovation in NEATG_A is the incorporation of a simple communication protocol between cells and the grid element they inhabit. The protocol consists of a basic ‘handshake’ between an individual cell and the grid element it inhabits. A signature is defined for each cell that includes a mix of genomic and phenotypic information. The genomic information includes data from all the genes in the cell. The phenotypic information includes the metabolic demands of the cell and the cell lifetime. The information is concatenated to form a single string signature. For grid elements the signature is defined as the signature of an untransformed (*Normal*) cell. The handshake consists of a comparison of signatures and the calculation of the Levenshtein distance (i.e., a numeric measure of the differences between the signatures) (Levenshtein, 1966). This distance metric counts the number of additions, deletions and point differences between two strings. In the case where signatures match (i.e., an individual *Normal* cell has the same signature as the grid element it inhabits) the distance is zero. In the case of *Malignant* cells with one or more genomic mutations or phenotypic alterations the distance metric is greater than zero, and the greater the degree of difference between the *Normal* cell the greater the numeric value of the distance. Cell-tissue communicative dysfunction is defined, therefore, as those instances where the distance > 0, in other words when the grid level and the cell level do not share the same signature.

Table 1 shows a number of examples of cell data structures, the corresponding cell signature and the Levenshtein distance between the *Malignant* cells and the *Normal* cell (row 1). Digits indicated in red in the cell signature are point changes in relation to the *Normal* cell signature.

**Table 1:** Cells, cell signatures, and distance.

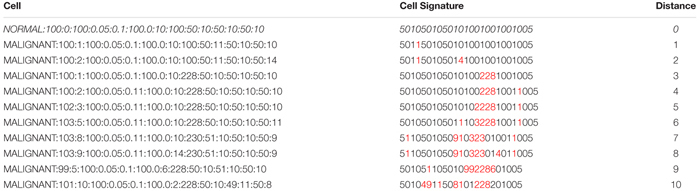

*Note that all distances are measured in comparison with the Normal cell in row 1. Cell signature digits in red indicate changes in comparison to the Normal cell. In the last two rows changes include digit deletions.*

In order to model the impact of communicative dysfunction, and also to assess the impact of communicative reprogramming, the NEATG_A system includes a system-level parameter, which we have termed *Tolerance*, which is a measure of the degree of tolerance or permissiveness of signature differences. A healthy system is defined as one where there is zero tolerance (Tol = 0) for differences during the handshake. In other words the *Tolerance* acts as a threshold on the value of Distance allowed in the system. The greater the degree of tolerance the greater the probability that mutated cells are able to proliferate successfully. By varying the degree of *Tolerance* at run-time the NEATG_A system is able to model impacts of communicative dysfunction and reprogramming on tumor growth.

**FIGURE 1:**
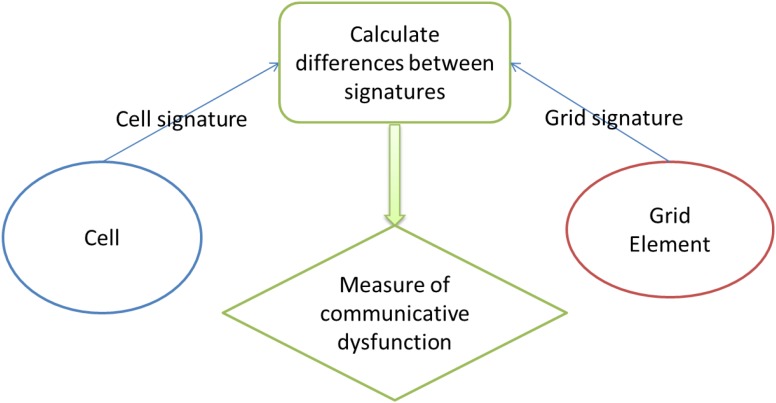
Handshake protocol.

The handshake protocol is shown in Figure 1.

As with the base NEATG system all cells undergo a simple cell fate program:

Quiescent>Dividing>Apoptotic>To Be Cleared

**FIGURE 2:**
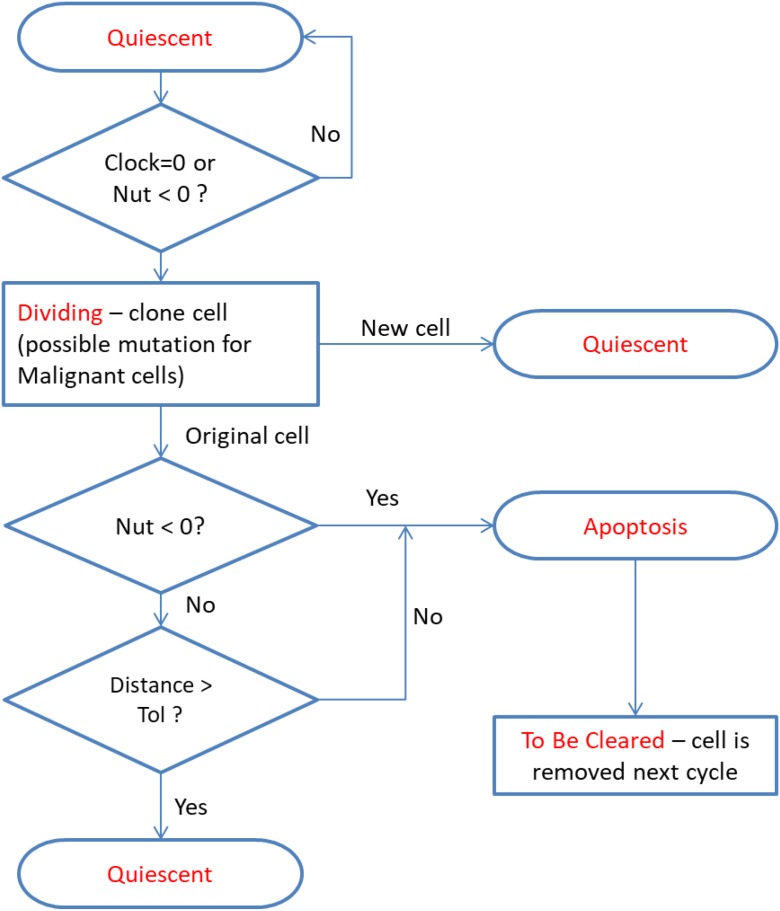
Cell fate program – note the same cycle is used for both *Malignant* and *Normal* cells. At cell division *Malignant* cells may probabilistically undergo a mutational event.

In this system *Quiescent* is simply defined as the state the cell is in while it is not under-going division or apoptosis – it implies viability and therefore both *Malignant* and non-*Malignant* cells are considered *Quiescent*. The cell fate program is shown in Figure 2 – note that cell states are indicated in red.

**FIGURE 3:**
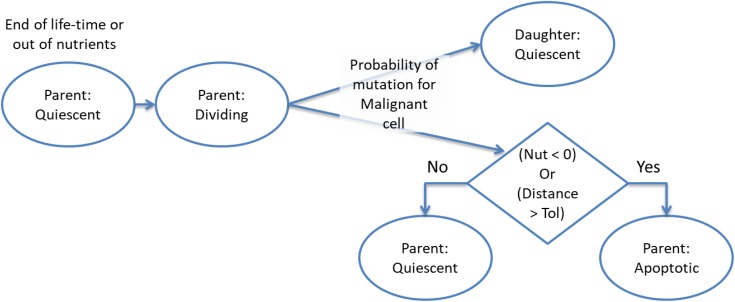
Cell division and apoptosis.

There are two aspects of the cell fate program which warrant particular attention. The first point is that cells may transition from *Dividing* to *Apoptotic* when they have reached the end of their lifetime, which may have been accelerated for cells poorly adapted to their local environment, or when their metabolic demands exceed the availability of nutrient supply. Cell division will result in a new *Quiescent* daughter cell (which may be *Normal* or *Malignant*, depending on the type of the parent cell). The parent cell may move to the *Apoptosis* state if it is out of nutrients or the cell exceeds the allowed degree of mutational change (i.e., the handshake protocol is invoked). In the homeostatic situation most cases cell of division do not lead to Apoptosis and both parent and daughter cells are *Quiescent*. However, when there is a high degree of nutrient stress, over-population and competition due to encroaching *Malignant* cells then division may lead to *Apoptosis* for the parent cell. This process is shown in more detail in Figure 3.

The second key point is that the only difference between cells flagged as *Normal* or *Malignant* is that during cell division *Malignant* cells may probabilistically undergo mutation. A mutation event is triggered if a random number in the range 0–1 is generated and is below the level of the mutation rate (which is a model parameter in the range 0–1, with typical values being in the order of 0.05). In grid elements which are overcrowded there is also a probability that a *Malignant* cell may migrate to a neighboring grid element, again using random number generation to assess the probability of an invasion event occurring. Cell division therefore can both increase the overall number of cells, including mutable *Malignant* cells when there is communicative dysfunction, and it can also lead tumor cell invasion.

The NEATG and NEATG_A systems are designed to test different treatment strategies and include the functionality to define new treatment protocols. In this work four such strategies are explored. The first is a *No Treatment* strategy in which tumor growth is allowed to proceed unchecked. This strategy acts as a baseline to explore the impact of changing the degree of communicative dysfunction in order to assess the impact on tumor growth. The *Chemo Treatment* strategy assesses the impact of a single high-dose treatment with a generic cytotoxic agent. This is a strategy that was also used in the original NEATG model. The *Communicative Reprogramming* strategy is a new protocol developed for NEATG_A. This treatment strategy is used to change the *Tolerance* level during a model run such that we can assess the impact of tumor growth when communicative reprogramming changes the degree of communication dysfunction after tumors are established. Finally, the combination of *Chemo* and *Communicative Reprogramming* allows the system to model the combination of cytotoxic treatment with reprogramming communicative dysfunction.

All experiments were performed on a laptop with an Intel i7-5500U 2.40 GHz processor, 12 GB of RAM and running 64-bit Microsoft Windows 10. The NEATG_A system was executed using a Java hot-spot 64-bit server VM/JRE 1.8. In terms of execution time the key determinants of performance are the size of the grid and the number of iterations per run. The algorithm displays *O(n^2^)* performance with respect to grid size and *O(n)* performance with respect to number of iterations. The mean and standard deviation of elapsed time per 1000 iterations is shown in Table 2.

**Table 2:** Mean and standard deviation of elapsed run-time per 1000 iterations, in seconds, for different grid sizes.

Len/width	Mean elapsed time (seconds)	SD elapsed time (seconds)
25	20.1	3.7
50	81.2	12.5
75	155.3	8.5
100	314.8	18.5


## Results

### No Treatment

In this first series of experiments the intention is to show the tumor growth dynamics under different levels of *Tolerance*. A 50 × 50 grid element is used, with each element of the grid populated with five *Normal* cells. At time = 0 a single *Malignant* cell is seeded in the central grid element and each experiment was allowed to run for 3000 iterations. Both *Normal* and *Malignant* cells have the same three-gene structure as previously used in the NEATG experiments as they are known to consistently generate viable tumor growth. The *Tolerance* value was varied from 0 to 9, and the system run 10 times for each value and the data averaged for analysis. All tests of significance use Student’s *t*-test.

**FIGURE 4:**
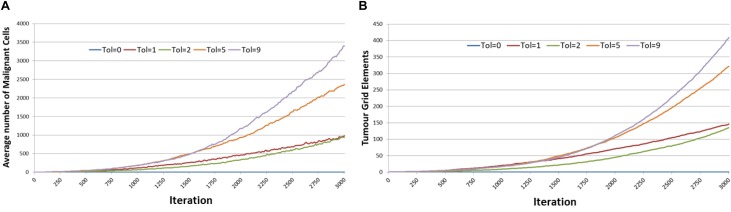
Impact of Tolerance on rate of tumor growth. **(A)** Change in Malignant cell counts over time for different values of Tolerance. **(B)** Change in number of Grid Elements invaded by tumor over time for different values of Tolerance.

Figure 4 shows the averaged results for different values of *Tolerance* – note that in the interests of clarity not all values of *Tolerance* have been shown, and mean values only are reported. Tumor growth can be assessed either as the simple count of *Malignant* cells at each time point, as shown in Figure 4A, or as the spatial distribution of *Malignant* cells (i.e., the number of grid elements which have been invaded by *Malignant* cells), as shown in Figure 4B. It is apparent from both these figures that when Tol = 0, i.e., when there is no communicative dysfunction in the system, that tumor growth does not take place. As the system becomes more permissive, that is as the *Tolerance* value increases, the rate of tumor growth clearly increases. The relationship is not linear, for example the data shows that in this dataset tumor growth when Tol = 2 is slightly slower than when Tol = 1, but as this is an evolutionary system there are instances where particularly beneficial mutations may emerge which can develop into aggressive tumors.

**FIGURE 5:**
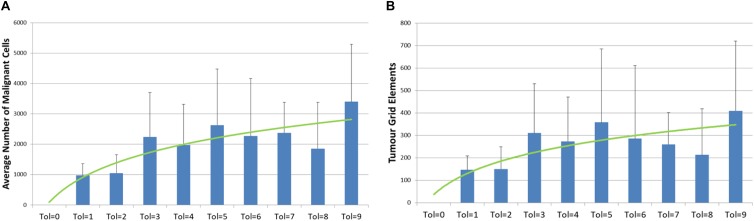
Relationship between tumor growth and Tolerance value. **(A)** Number of Malignant cell counts at 3000 iterations for different values of Tolerance (mean and standard deviation). **(B)** Number of Grid Elements invaded by tumor at 3000 iterations for different values of Tolerance (mean and standard deviation).

However, if we compare the final values of *Malignant* cell counts and invaded grid elements at the end of system runs (at iteration 3000), then we can see a clear relationship between tumor growth and degree of communicative dysfunction, as shown in Figure 5. Note also the standard deviation showing the variance between different runs of the system for the same parameters.

The relationship between *Tolerance* value and the number of *Malignant* cells at 2000 iterations is statistically significant, *P* = 0.007. Also significant is the rate of growth between iteration = 1100 and 1500 and *Tolerance* value, *P* = 0.001, and between iteration = 1500 and 2000, *P* = 0.000001. These time periods are used in the experiments that follow as they are sufficient to allow tumor growth to occur before treatment interventions take place and yet are sensitive to the *Tolerance* value.

**FIGURE 6:**
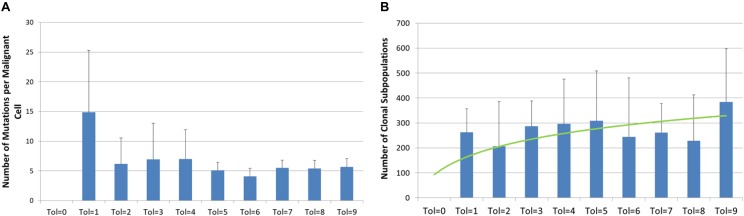
Genetic heterogeneity and Tolerance value. **(A)** Number of mutations per malignant cell at 3000 iterations for different values of Tolerance (mean and standard deviation). **(B)** Number of clonal subpopulations at 3000 iterations for different values of Tolerance (mean and standard deviation).

The *Tolerance* value also has some effects on the degree of genetic heterogeneity, as shown in Figure 6. In Figure 6A we see the average number of mutations per *Malignant* cell at the end of the 3000 iterations. It is clear that in the case of Tol = 1 the *Malignant* cell population has undergone a far higher degree of evolutionary change than the *Malignant* cells in more *Tolerant* conditions. In contrast we see in Figure 6B that there is a trend to a higher number of clonal subpopulations (i.e., distinct populations of *Malignant* cells sharing the same genomes) in more *Tolerant* conditions. The data is suggestive that there is a greater evolutionary pressure for low values of *Tolerance* but that this evolutionary pressure does not necessarily lead to greater intra-tumor heterogeneity.

**FIGURE 7:**
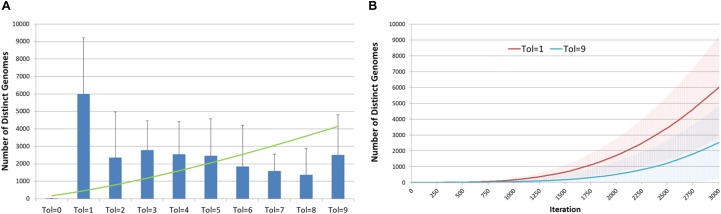
Genomic evolution and Tolerance. **(A)** Number of distinct genomes at 3000 iterations for different values of Tolerance (mean and standard deviation). **(B)** Evolution of distinct genomes for Tol = 1 and Tol = 9 (mean and standard deviation).

Another view of the degree of evolutionary change and the relationship with *Tolerance* is shown in Figure 7. Figure 7A shows the cumulative number of distinct genotypes which have existed through the lifetime of the system, including all clonal subpopulations that have become extinct. This is in effect a measure of the rate of evolutionary change in the system, and it clearly indicates that Tol = 1 has a higher rate of change than more *Tolerant* conditions. In Figure 7B we show the secular trend for Tol = 1 and Tol = 9, showing that relative rates of change. Note also the degree of variance indicated by the error bars.

**FIGURE 8:**
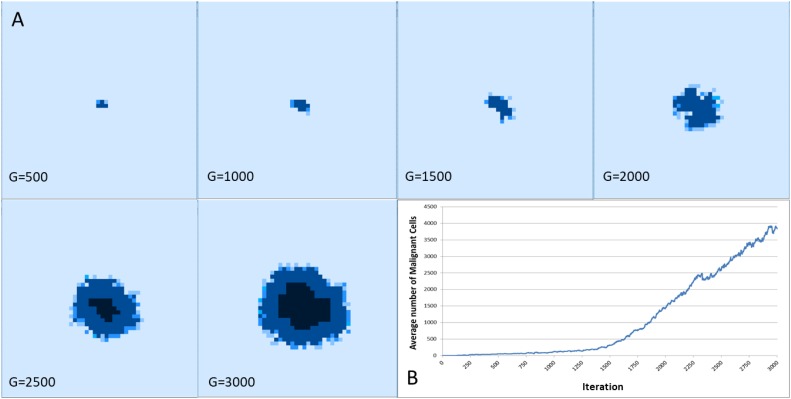
**(A)** Spatial distribution of tumor growth over time. Note the emergence of necrotic areas in the tumor mass (denoted in black). **(B)** The corresponding growth curve showing increase in Malignant cell counts.

Figure 8 shows the spatial distribution of tumor growth for one representative run of the system. In this example Tol = 9 and the figure shows an aggressive tumor mass that expands over time.

### Cytotoxic Treatment

Having established that increasing communicative dysfunction is associated with more aggressive tumor growth, the next series of experiments will assess the relationship between cytotoxic treatment and communicative dysfunction. The same parameters will be used as in the previous experiments though the system will be run for only 2000 iterations as we know that by this stage tumors are generally well-established. The treatment strategy used in these experiments is based on the idea of maximum tolerated dose chemotherapy. Treatment is initiated at iteration 1500 and is applied for 25 iterations. The treatment induces apoptosis in cells close to cell division, with *Malignant* cells having a greater vulnerability, although there is some level of ‘collateral’ damage to *Normal* cells (i.e., some *Normal* cells also undergo apoptosis due to the treatment). Full details of this treatment strategy are described in Pantziarka (2016), and in this case the ratio of *Malignant* to *Normal* cells that undergo apoptosis through this treatment is 5:1. Experiments were repeated 20 times and the mean value used for the analysis.

**FIGURE 9:**
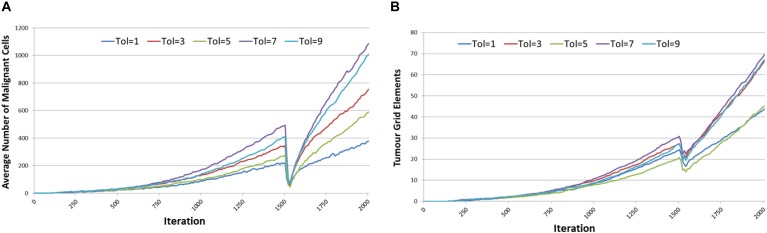
Tumor response to cytotoxic treatment. **(A)** Impact of cytotoxic treatment on Malignant cell counts for different values of Tolerance. **(B)** Impact of cytotoxic treatment on tumor invasion for different values of Tolerance.

The results are shown in Figure 9. For both *Malignant* cell counts and for tumor invasion the initiation of cytotoxic treatment causes a sharp decrease in cell numbers, in line with real tumor response to cytotoxic chemotherapy. However, following the cessation of treatment there is a recovery of tumor growth. It is clear that there is a relationship between communicative dysfunction and this regrowth rate – the greater the value of *Tolerance* the more aggressive the rare of regrowth.

**FIGURE 10:**
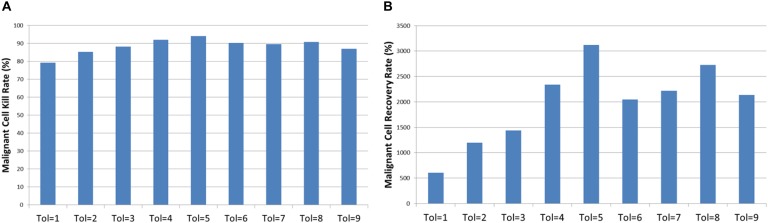
Tumor kill and recovery rates. **(A)** The tumor kill rate for different values of Tolerance. **(B)** The tumor regrowth rate for different values of Tolerance.

Two important metrics when assessing cytotoxic therapy are the kill rate and the tumor regrowth rate. The first is the percentage of the *Malignant* cells that die off due to the cytotoxic treatment – in our example this is computed as the percentage difference in *Malignant* cell counts at treatment initiation (iteration = 1500) and the first time point immediately after cessation of treatment (iteration = 1526). The tumor regrowth is defined here as the percentage difference in *Malignant* cells count from cessation of treatment (iteration = 1526) to 400 iterations after treatment cessation run (iterations = 1926). The results are shown in Figure 10, the relationships between *Tolerance* and kill rate and *Tolerance* and recovery rate are both significant at *P* = 0.001. Also significant is the relationship between kill rate and recovery rate at *P* = 5.4 × 10^-9^.

### Anakoinosis – Communicative Reprogramming

A key element of the anakoinosis concept is the communicative reprogramming through the use of a cocktail of repurposed non-cancer drugs to modulate the biology of the entire tumor system rather than focus purely on the cancer cells (Hart et al., 2015; Walter et al., 2017). Given that we have shown that communicative dysfunction, as defined here by *Tolerance* > 0, is associated both with tumor growth and tumor regrowth following cytotoxic treatment, the next series of experiments investigated the effect of communicative reprogramming on tumor growth.

The model run parameters are as in the previous section, with the exception that a *Tolerance* value of Tol = 9 is used as the background rate. As was shown above, this level of communicative dysfunction is associated with robust tumor growth over a period of 2000 iterations. The treatment strategy is initiated at iteration 1500, when communicative reprogramming takes place and the *Tolerance* is reduced, as we would expect with anakoinosis treatment, to the range Tol = 0 to Tol = 8. Each experiment was repeated 10 times and the mean of the results used in the analysis. Note that treatment in these experiments was for iterations = 100, a value that empirical testing had shown was associated with robust responses.

**FIGURE 11:**
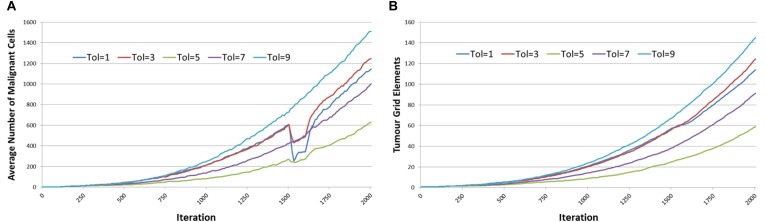
Tumor response to anakoinosis. **(A)** Impact of anakoinosis treatment on Malignant cell counts for different values of Tolerance. **(B)** Impact of anakoinosis treatment on tumor invasion for different values of Tolerance.

**FIGURE 12:**
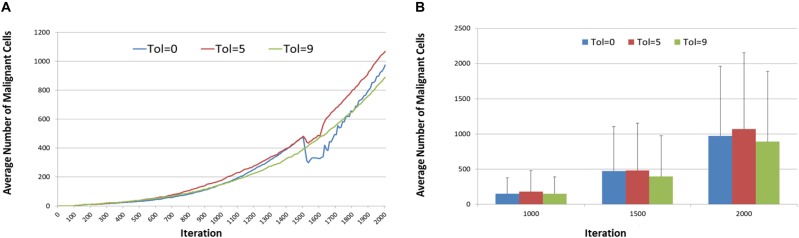
Anakoinosis treatment for 50 runs. **(A)** Mean pre-treatment growth rate for 50 runs. **(B)** Mean and standard deviation of Malignant cell counts after 50 runs at 1000, 1500, and 2000 iterations.

Figure 11 shows the tumor response, in terms of *Malignant* cell counts and tumor invasion, of anakoinosis – the communicative reprogramming such that *Tolerance* is reduced from Tol = 9 to lower *Tolerance* for the duration of treatment, that is between iterations 1500 to 1600. The *Tolerance* value is reduced in a step-change and remains at the lower level until the end of treatment, at which point it is increased again in a step-change back to Tol = 9. Note that the control case, where *Tolerance* is not reverted but remains at Tol = 9 is also shown. There are differences in growth rates prior to initiation of treatment at iteration = 1500, but as this is an evolutionary model we would expect some variation. Cell counts at iteration = 500, 1100, and 1500 were uncorrelated with the treatment schedule and essentially chance artifacts that are not significant. Furthermore, an additional set of experiments was performed for 50 runs for three values of treatment to confirm that the pre-treatment growth rates converged, as shown in Figure 12A,B.

It is the reduction in cell numbers during and after treatment which are of importance and clearly the responses in terms of the number of *Malignant* cells are quite strong (Figure 11A), however there is a less clear effect in terms of a reduction in tumor invasion as shown in Figure 11B. It is also apparent from Figure 11A that recovery in *Malignant* cell numbers emerges very quickly after treatment ends at iteration 1600, in many cases before the treatment has ceased.

**FIGURE 13:**
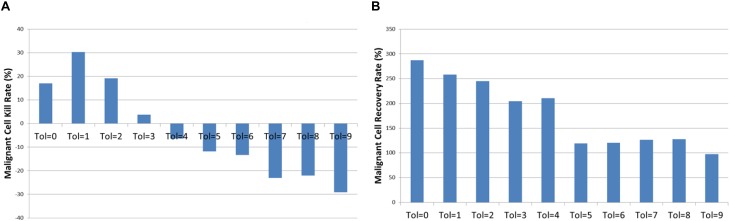
Tumor kill and recovery rates following anakoinosis. **(A)** The tumor kill rate for different values of Tolerance after anakoinosis treatment. **(B)** The tumor regrowth rate for different values of Tolerance after anakoinosis treatment.

Examination of the mean kill rate (difference in *Malignant* cell counts between iteration = 1500 and iteration = 1601), shows that in fact with the exception of reversion to the lowest levels of communicative dysfunction (Tol = 0 to Tol = 2) the treatment did not lead to a reduction in tumor growth, as shown in Figure 13A. Paradoxically, the mean recovery rate is higher for the lower values of *Tolerance* following the cessation of treatment, as shown in Figure 13B. However, while the rate increases rapidly, the final cell counts are lower for lower values of *Tolerance* during treatment.

The relationship between the reverted *Tolerance* value and *Malignant* cell kill rate is highly significant, *P* = 2.2 × 10^-23^, and the relationship between kill rate and recovery is also highly significant at *P* = 3.8 × 10^-7^.

### Combination Treatment

In the final set of experiments we explore the strategy of combining cytotoxic treatment with communicative reprogramming. In this series of experiment we will assess the impact of applying the cytotoxic treatment in parallel with reverting communicative dysfunction from Tol = 9 to Tol = 0. The parameters for the cytotoxic treatment are as before. In addition in this series of experiments the impact of increasing the anakoinosis treatment period, from 100 to 500 iterations is assessed. The duration of the model runs is extended to iteration = 2500 so that the impact of the longer treatment period can be assessed.

**FIGURE 14:**
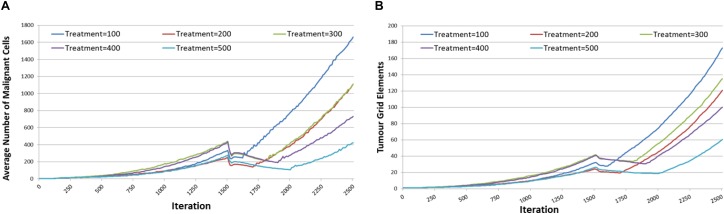
Tumor responses to combination treatment. **(A)** Impact of combination treatment on Malignant cell counts for different treatment duration. **(B)** Impact of combination treatment on tumor invasion for different treatment duration.

The results are shown in Figure 14, and it is apparent, both in terms of *Malignant* cell counts and grid elements, that the initial sharp drop in cell counts due to the combination of cytotoxic treatment and communicative reprogramming is followed by a slower decline in cell numbers. Tumor regrowth commences only after the cessation of all treatments. As we would predict, the longer treatment period is associated with longer responses to treatment.

**FIGURE 15:**
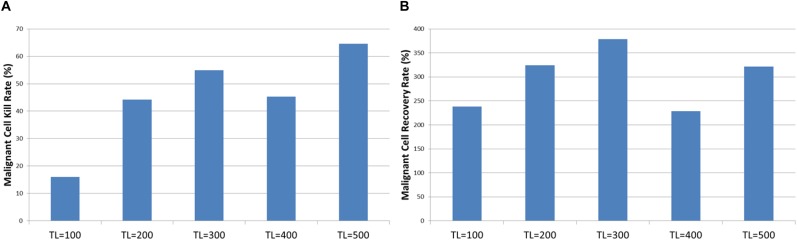
Tumor kill and recovery rates after combination treatment. **(A)** The tumor kill rate for different duration of combination treatment. **(B)** The overall Malignant cell growth rate for different duration of combination treatment.

The tumor kill and recovery rates are shown in Figure 15, and these reflect the same findings in that longer treatment periods are strongly associated with greater kill rates and reduced recovery rates. The relationship between kill rate and treatment length is significant, *P* = 1.1 × 10^-9^, as is the relationship between kill rate and recovery, *P* = 4.2 × 10^-4^.

## Discussion

Both NEATG and NEATG_A are non-physiological agent-based evolutionary models. While they exhibit complex behaviors that are analogous to real tumor systems they do not infer specific biological pathways or molecular signaling. Agent-based evolutionary models are an ideal methodology for investigations into biological systems at the cell level. Unlike more traditional mathematical/statistical models, agent-based models are designed to investigate the interactions between many individual cells rather than treating all cells as an amorphous mass. Recent publications in oncology have investigated the Warburg effect (Shan et al., 2018), drug-radiation interactions in the tumor-microenvironment (Mao et al., 2018) and an investigation into tumor response to PD1 and PDL1 inhibition (Gong et al., 2017). There are many others, demonstrating that this modeling technique is able to generate new insights and results in biologically relevant contexts. In the case of NEATG some of the mechanisms described in the model, particularly related to the role of cell competition and the pro-tumor impact of apoptosis, have subsequently been found to be in accord with the role of Myc-mediated cell competition in cancer initiation and progression (Di Giacomo et al., 2017b).

**FIGURE 16:**
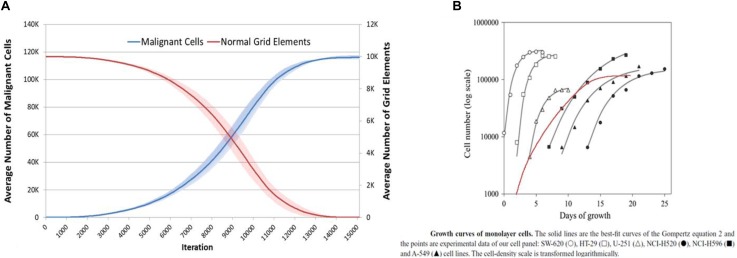
NEATG_A data comparison to real tumor growth. **(A)** Long-term growth, for l5000 iterations, of Malignant cells and concomitant reduction in non-tumor grid elements (mean and standard deviation). **(B)** Growth of monolayer cells from human cancer cell lines (reproduced from Castro et al., 2003), superimposed in red Malignant cell growth from **(A)** (red line) scaled and transformed to compare to human cancer cell lines.

Data previously published showed that the NEATG model results were similar to *in vitro* results for a number of cancer cell lines (Pantziarka, 2016). Figure 16, shows the results produced by the NEATG_A model in comparison with growth data from a published panel of human cancer cell lines (Castro et al., 2003). These results (produced with Tol = 9, no treatment and run for 15000 iterations), shows that as with the previous version of the model, NEATG_A cancer cell growth dynamics recapitulate features from *in vitro* laboratory data. This data also suggests that with high values of *Tolerance* this version of the model produces results in line with the non-anakoinosis version of the model.

In the case of this work, NEATG_A is a significant advance on the original NEATG model. It has incorporated the idea of communicative dysfunction and communicative reprogramming into the model. The model itself is agnostic as to the specific biological features of this cell-tissue communication system, although it incorporates both genotypic and phenotypic features into a handshake signature between cells and tissue. The results show that this cell-tissue communication system acts as an intrinsic non-cell autonomous anticancer mechanism. This enhancement of the model therefore increases the biological relevance and incorporates additional mechanisms which are of great importance in mediating responses of real tumors to treatments. In particular, the interaction between cell-mediated and cell-autonomous processes reflects more closely the underlying biology of cancer.

There is some evidence to support such a finding in biological systems, for example Bussard and Smith report experiments in which cancer cells transplanted into non-cancer mammary tissues are reverted from their cancer phenotype (Bussard and Smith, 2012). Indeed there is some interest in the idea of a pre-cancerous niche, whereby cancer initiation is associated with chronic inflammation, tissue injury or some other cause of tissue dysfunction (Pantziarka, 2015). Multiple lines of evidence point to the importance of the host tissue environment in the formation of metastases (Kaplan et al., 2006; Barcellos-Hoff et al., 2013). Clearly such tissue dysfunction, which is associated with many of the “hallmarks of cancer” (Hanahan and Weinberg, 2011), involves multiple cell-dependent and independent processes. Therefore, a handshake signature that combines both genomic and phenotypic data is supported by a strong biological rationale.

Intra-tumor heterogeneity is an important feature of cancer and the focus of intense interest (McGranahan and Swanton, 2017), particularly as it may relate to response to cancer treatments (Jamal-Hanjani et al., 2015; Gatenby and Brown, 2017). As shown in Figure 6, 7, the model exhibits the evolutionary development of sub-clonal expansion and an increase in mutational load. Furthermore the data shows that *Tolerance* is an important evolutionary factor – low *Tolerance* (Tol = 1) acts on selection and therefore drives a greater degree of evolutionary change (in terms of mutational load and the accumulated number of distinct genomes expressed during 300 iterations of the system) than more *Tolerant* systems. In this system the *Malignant* cells try to adapt to the low *Tolerance* conditions by rapid rates of change. However, we may note that this greater rate of mutational change does not necessarily lead to greater rates of tumor growth. In fact despite the greater rate of mutational burden shown when Tol = 1, the growth of *Malignant* cell counts and tumor invasion is highest when *Tolerance* is higher. Indeed, as shown in Figure 6B, the greatest number of clonal subpopulations exists when Tol = 9, suggesting that under lower *Tolerance* levels many of the clonal subpopulations do not survive.

Of course in physical systems the degree of *Tolerance* would not be static and may co-evolve with the growth of clonal subpopulations. We may speculate that a cancer may first develop in situations of low *Tolerance* in which a high degree of mutational change takes place, but that as the cancer develops the degree of *Tolerance* increases thereby leading to rapid expansion of some of these clonal subpopulations.

### No Treatment Results

The initial series of experiments, using the *No Treatment* scenario, are broadly in line with the results from the NEATG model, with the exception of the cases where *Tolerance* = 0. These results consistently show that the implanted *Malignant* cells are not able to establish viable tumors in a ‘healthy’ tissue environment. However, when there is a degree of permissiveness, as set by *Tolerance* > 0, then implanted *Malignant* cells proliferate and form invasive tumors. The relationship between communicative dysfunction and the degree of tumor growth, as shown in Figure 5, appears to be broadly logarithmic. Why would we not expect a linear relationship? A greater *Tolerance* value would translate into an increased evolutionary search space for *Malignant* cells – but this increased capacity for mutational change also means that there is a greater probability for mutations that are either ‘silent’ or that reduce the fitness of individual *Malignant* cells. Therefore we may expect to see some diminishing returns as the *Tolerance* value increases. We should also note that this is a stochastic model and that in some runs particularly good or bad evolutionary trajectories emerge, with corresponding high or low rates of tumor growth. For example, as clearly shown in Figure 5, the results for tol = 5 are higher than expected, while for tol = 8 the figures lower.

### Treatment Results

The first treatment strategy to be assessed is the *Chemo* strategy, which is designed to be analogous to high-dose cytotoxic therapy. The treatment parameters, including treatment period (25 iterations), were based on previous results that have been shown to induce a significant reduction in *Malignant* cell numbers and the degree of invasion (as measured by the number of grid elements which predominately contain *Malignant* cells). In this series of experiments it is not the treatment that was varied but the degree of communicative dysfunction as defined by the *Tolerance* value. The results show statistically significant relationships between the *Tolerance* value and both tumor kill rate (the proportion of *Malignant* cells killed by the end of treatment) and the rate of tumor regrowth.

In particular it is the relationship between the recovery rate and the degree of *Tolerance* which is important here. The implication is that resistance to therapy, in the form of fast tumor regrowth following chemotherapy, may be related to the level of tissue dysfunction rather than being driven entirely by the characteristics of cancer cells. This phenomenon, which has been termed environment-mediated drug resistance (Meads et al., 2009), is clearly of clinical concern. There is evidence that chemotherapy itself may exacerbate tissue dysfunction and therefore induce further resistance to treatment (Vyas et al., 2014). For example, high rates of apoptosis in breast cancer has been shown to be associated with a worse prognosis (Nishimura et al., 1999), and a possible prognostic marker in circulating tumor cells (Jansson et al., 2016). Apoptotic cell death is clearly not a ‘silent’ process, with multiple impacts on surrounding cells, tissues and immunological responses (Gregory and Pound, 2011; Wang et al., 2013; Labi and Erlacher, 2015; Lauber and Herrmann, 2015). In the context of anakoinosis, the ‘phoenix rising phenomenon’ is especially pertinent in that caspase-3-dependent apoptosis induced by cytotoxic chemotherapy activates the COX-2 pathway, thereby promoting prostaglandin-E2-mediated proliferation of surviving cells (Fogarty and Bergmann, 2015).

In the next series of experiments the focus was on reverting communicative dysfunction, in the form of changing the *Tolerance* value following the successful seeding of a tumor mass. The background *Tolerance* value was set at Tol = 9, which the previous experiments have shown is associated with aggressive tumor growth, and then at initiation of treatment at iteration 1500 this *Tolerance* value was reduced in the range Tol = 0 to Tol = 8. The results, in Figure 11, show that reducing *Tolerance* from a high value to a low value can slow the rate of tumor growth and, in some cases, cause a degree of tumor regression. It also shows that while Tol = 0 can stop the formation of tumors, once a tumor is well-established reverting to Tol = 0 is not sufficient to completely remove all *Malignant* cells. It is also clear that only large reductions in *Tolerance* value can induce tumor regressions. Both Figure 11A, 12A therefore illustrate the same behavior – that reducing *Tolerance* from a high level (Tol = 9) to a low-level (between Tol = 0 and Tol = 5) induces growth arrest or regression for the duration of the low *Tolerance* period only but that when treatment ends and *Tolerance* returns to a high level tumor growth recommences and indeed may be more aggressive than prior to treatment. This finding has important implications in terms of treatment protocols for cancer patients undergoing anakoinotic treatments.

Also of note is that tumor regrowth following cytotoxic treatment, as shown in Figure 9, is more aggressive than the regrowth following communicative reprogramming, Figure 11. We may speculate that the former reduces tumor growth through inducing artificially high rates of apoptotic cell death, while the latter reduces tumor growth through tissue-intrinsic anticancer mechanisms.

These results suggest that patients treated with medicines to address systemic communicative dysfunction – in other words drugs for communicative reprogramming – would benefit more when the degree of normalization is highest. Further, it suggests that treatment with these drugs alone may not be sufficient to induce complete regressions. Finally, cessation of treatment may be followed be a rapid recurrence of disease. Data from a Phase II clinical trial of communicative reprogramming in non-curative hepatocellular carcinoma provides support for this model finding (Walter et al., 2017). If we accept that serum concentration of C-reactive protein (CRP) is a proxy for communicative dysfunction this study showed both that pre-treatment level was correlated with survival and, as importantly, that reduction in CRP during treatment was also predictive of survival.

In the final round of experiments the intention was to assess the combination of chemotherapy and communicative reprogramming. Additionally, given that all treatments have shown tumor regrowth following cessation of therapy, this series of experiments assessed the impact of extending the treatment periods. In this combination one initial dose of cytotoxic treatment was modeled, for iteration = 25 as before, concurrently with communicative reprogramming for periods of iteration = 100 to iteration = 500. The rationale for this approach is that increasing the duration or intensity of chemotherapy imposes unacceptable toxicity in patients, while communicative reprogramming using repurposed drugs is relatively non-toxic and more tolerable.

**FIGURE 17:**
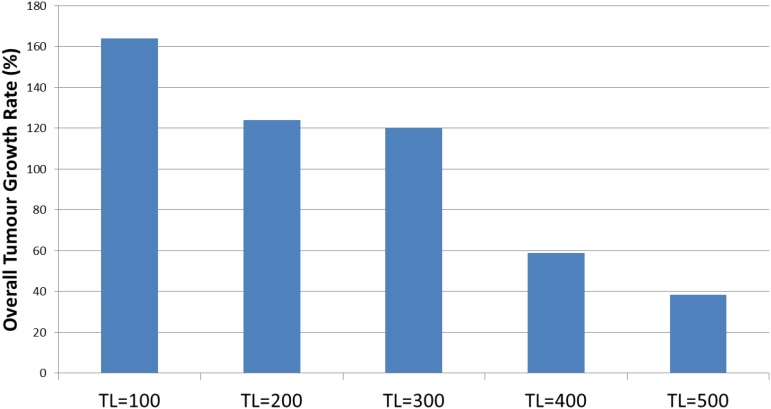
Growth at iteration = 400 following combination treatment.

Results in Figure 14 show that the combination treatment does induce a rapid reduction in *Malignant* cell count and subsequent tumor regrowth. However, the longer treatment periods are associated with a greater level of tumor cell kill and a slower rate of recovery. Indeed, calculation of the growth rate at iteration = 400 following cessation of treatment shows a very strong relationship, *P* = 7.7 × 10^-5^, with treatment length, as shown in Figure 17.

This result clearly supports the use of long-term treatments using the anakoinosis approach of communicative reprogramming with non-toxic therapies. It also suggests that the use of this approach with chemotherapy may also have some utility. A possible biological rationale is that DNA damaging drugs can elicit a signal response [DNA damage response (DDR)] able to modulate cell-autonomous and cell-non-autonomous processes, possibly driving cells toward a caspase-independent apoptosis – in other words to induce a ‘silent’ apoptotic response mediated by the combination of chemotherapeutics and anakoinotic drugs (Bruni et al., 2018).

Perhaps surprisingly, little, if any, experimental evidence of the *long-term* effects of anticancer treatments on *in vitro* cancer cell systems is available, especially dealing with modulatory drugs. Therefore, it is not possible so far to fully assess the validity of the NEATG_A system on this issue. To explore further, we set up *ad hoc* experimental protocols, using cytotoxic, modulatory, and combination treatments on human tumor cell cultures. The very long experiments, that are still ongoing, have demonstrated that cytotoxic treatments drastically reduce cell number, as expected, but promote faster regrowth of cells with increased chemoresistance, in line with the phoenix rising model. Modulatory drugs induce a more gradual growth inhibition, but do not promote faster regrowth; on the contrary, proliferation reduction is even maintained after drug removal, provided the drugs were maintained for a minimum number of days (e.g., necessary to reprogram gene expression). Such experimental findings, now in the completion phase and soon to be submitted (Corsi et al., unpublished), strikingly match the NEATG_A results, reinforcing the strong predictive ability of the model, and suggesting mechanisms through which the “handshake” may modulate the response to drug treatment in cancer patients.

One of the key results from previous work with the NEATG model, and confirmed in this study, is that high rates of cell death are pro-tumor and linked to subsequent tumor progression. The clear implication of these results is that anakoinosis may be effective as a cancer treatment because it alters the communicative environment and interferes with the pro-tumor impact induced by high rates of chemotherapy-related apoptosis. A second implication is that cell competition, which is an emerging area of research in oncology, is also impacted by anakoinosis – again, changing the communicative environment through reprogramming alters the tumor system such that *Malignant* cells with high-levels of fitness due to mutational change are penalized rather than rewarded.

A key strength is that these results arise from the functional interactions of the elements modeled by the system, namely cells and tissues. The behaviors are emergent rather than pre-programmed or explicitly coded in the model. As such it is encouraging that several clinically relevant outcomes from clinical studies using the anakoinosis approach also emerge in these model results. Furthermore, it suggests that additional mechanisms related to cell death and cell competition may be relevant in this treatment approach.

This is a computational model at a level of abstraction that, at this stage of development, does not consider the relevant biological/molecular pathways that implement these behaviors in the patient or animal model. For example, it does not include data on the relative importance of immune response, angiogenesis, tumor architecture or other key factors. However, there may be scope to enhance the model to include more physiologically relevant pathways such that the impact of addressing these factors clinically can also be modeled. The handshake function that is included in NEATG_A in this study is still a very crude protocol; recent work, for example on cell competition and carcinogenesis, suggests that there is a more complex interplay at work (Di Giacomo et al., 2017a; Watanabe et al., 2018).

## Conclusion

Anakoinosis, or communicative reprogramming is an emerging treatment modality that uses a combination of non-cancer drugs with standard cancer treatments. To date this approach has not been subject to mathematical or computational modeling. Here we present results from a complex agent-based evolutionary computational model. The results are broadly in line both with clinical results and with the rationale behind the development of the anakoinosis approach. These positive results lend support to the treatment approach, particularly the long-term use of low-toxicity treatments that reduce the risk of treatment-induced accelerated tumor regrowth and the process of environment-mediated drug resistance.

Finally, these results predict that a combination of cytotoxic treatment and communicative reprogramming lead to a reduced tumor regrowth following cancer treatment. This strategy warrants further pre-clinical investigation.

## Data Availability

The datasets generated for this study are available on request to the corresponding author. The source code for the model is available for download at the Figshare repository: https://figshare.com/articles/NEATG_A/7856726.

## Author Contributions

PP developed the model and wrote the first draft of the manuscript. LG and AR contributed revisions to the text. All authors read and approved the submitted version.

## Conflict of Interest Statement

The authors declare that the research was conducted in the absence of any commercial or financial relationships that could be construed as a potential conflict of interest.
